# The searchbuildR shiny app: A new implementation of the objective approach for search strategy development in systematic reviews

**DOI:** 10.1002/cesm.12078

**Published:** 2024-06-11

**Authors:** Claudia Kapp, Naomi Fujita‐Rohwerder, Jona Lilienthal, Wiebke Sieben, Siw Waffenschmidt, Elke Hausner

**Affiliations:** ^1^ Department of Information Management Institute for Quality and Efficiency in Health Care Cologne Germany; ^2^ Department of Non‐Drug Interventions Institute for Quality and Efficiency in Health Care Cologne Germany; ^3^ Department of Medical Biometry Institute for Quality and Efficiency in Health Care Cologne Germany

**Keywords:** data mining, evidence synthesis, information storage and retrieval, natural language processing, review literature as topic, systematic reviews as topic, user‐centered design

## Abstract

**Introduction:**

One of the main tasks in information retrieval is the development of Boolean search strategies for systematic searches in bibliographic databases. This includes the identification of free‐text terms and controlled vocabulary. IQWiG has previously implemented its objective approach for search strategy development using a fee‐based text analysis software. However, this implementation is not fully automated, due to a lack of technical options. The aim of our project was to develop a text analysis tool for the development of Boolean search strategies using R.

**Methods:**

We adopt an incremental approach to software development, with the first goal being to develop a minimum viable product for the previously defined use cases. To create an interactive user interface, we use the shiny framework.

**Results:**

Our newly developed shiny app searchbuildR is a text analysis tool with a point‐and‐click user interface, that automatically extracts and ranks terms from titles, abstracts, and MeSH terms of a given test set of PubMed records. It returns searchable, interactive tables of free‐text and MeSH terms. Each free‐text term can also be viewed within its original context in the full titles and abstracts or in a user‐defined word window. In addition, 2‐word combinations are extracted and also provided as an interactive table to help the user identify free‐text term combinations, that can be searched with proximity operators in Boolean searches. The results can be exported to a CSV file. The new implementation with searchbuildR was evaluated by validating the text analysis results against the results of the previously used fee‐based software.

**Conclusions:**

QWiG has developed the shiny app searchbuildR to support the development of search strategies in systematic reviews. It is open source and can be used by researchers and other information specialists without extensive R or programming skills. The package code is openly available on GitHub at www.github.com/IQWiG/searchbuildR.

## 1 INTRODUCTION

Systematic reviews aim to inform evidence‐based decision‐making in health care. This type of review involves systematic information retrieval to identify as many relevant studies as possible without systematically missing relevant studies on the question of interest [1]. One of the tasks is to develop search strategies for searching bibliographic databases. Boolean search queries include free‐text terms and controlled vocabulary from a database‐specific thesaurus. They aim to identify precise word matches in citation metadata (e.g., title, abstract, keywords, controlled vocabulary, authors, publication year, journal, etc.).

The Institute for Quality and Efficiency in Health Care (IQWiG), the German health technology assessment (HTA) agency, bases its methods for developing search strategies [2, 3, 4] on established methods for developing and validating study filters [5]. This approach is also known as the “objective approach” [6] and unlike the purely conceptual approach it includes a set of relevant documents to identify terms [4, 7, 8]. Its strengths are that its transparency allows informed decisions to be made about including (or excluding) terms, and that it allows information specialists to work more independently [3]. With freely accessible tools now widely available text mining, has become increasingly popular for identifying search terms beyond the objective approach described by Lefebvre and colleagues [1, 3, 9, 10].

IQWiG has previously implemented the objective approach for search strategy development by Hausner et al. [3, 4] using the fee‐based text analysis software Wordstat [11]. However, this implementation is still iterative, that is, not fully automated, due to a lack of technical options. Furthermore, the workflow in Wordstat is quite complicated and required extensive expertise about the text‐mining process. The software is also expensive. As an alternative, freely available (open source) and more sophisticated tools are increasingly being used by information specialists [8, 9]. For example, PubReMiner [12] and the R‐based litsearchr [13] are two popular text‐mining tools. R has become an established programming language in statistical computing, including text analysis. Although the Python programming language is also useful, in our opinion R is more beneficial because the introduction of the shiny framework [14] by Rstudio (now Posit) [15] in 2012 has enabled easy implementation of graphical user interfaces for HTML‐based web applications (=shiny apps), without requiring advanced knowledge of programming languages other than R. R‐based methods are being further developed and have been presented in scientific publications (e.g., Grames et al. [13]) and at meetings (e.g., at the Evidence Synthesis and Meta‐Analysis in R Conference, ESMARConf [16]). IQWiG has therefore developed an R package and shiny app, searchbuildR, which allows easy implementation of customized solutions as well as validation of methodological procedures. It aims to make the text‐mining workflow for search term identification of the objective approach freely available and easily replicable as, for example, requested by Adam [8] by providing ranked lists of candidate search terms.

To further refine and automate the objective approach, the aim of our project was to develop a text analysis tool for the development of Boolean search strategies in systematic reviews using R. We published the project protocol a priori on Zenodo [17] and presented a proof‐of‐concept version of searchbuildR at ESMARConf 2023 [18] and a preliminary version 0.0.12. at the annual workshop of the European Association of Health Information and Libraries (EAHIL) [19].

## 2 MATERIALS AND METHODS

### 2.1 Use cases

Before the start of the project, we held an internal workshop where IQWiG information specialists defined their requirements for new information retrieval tools and discussed established workflows for search strategy development. The aim was to identify the main requirements for identifying candidate terms using a text analysis approach. It was stated that the new tools should make the work easier, while maintaining a high level of quality. To achieve this, the tools should be flexible and compatible with existing tools and workflows. The previously established core functions were still considered to be valid: single word frequency, 2‐word frequency, frequency metrics by document and separately for free‐text terms (title/abstract) and controlled vocabulary terms, statistical comparison with a basic set, and export of the results to Microsoft Excel. The Wordstat workflow to implement them consisted of five steps: (1) import of a test set, (2) data preparation, (3) text frequency analysis and statistical analysis, (4) analysis of word combinations and (5) interactive display of terms in their original context. This workflow provided the basis for defining a minimum viable product (MVP). Additional functions would be useful (see Section 4 Discussion).

### 2.2 Text analysis

The objective approach to search strategy development involves the analysis of simple word frequencies and word combinations that are derived from a set of known relevant records (=test set) [3, 4]. As a result the tool provides the user with a ranked list of candidate search terms. The identification of candidate search terms is based on statistical overrepresentation [4]. The probability of the occurrence of each term in the test set is statistically compared to a basic set represented by a set of randomly selected PubMed records (=population set). The aim is to identify not only the most sensitive terms but also precise and overrepresented terms (=candidate terms) that can distinguish relevant records from the population set. Wordstat offers a feature that calculates a *z*‐score for each term in the test set, which serves as an estimate of term overrepresentation. In previous projects, we empirically determined a cut‐off of *z* ≥ 20 to identify candidate terms [4]. According to the Wordstat support team, their *z*‐scores are based on a normal approximation of a binomial distribution under a random sampling model. We implemented the statistical comparison in R as a binomial test to calculate the *z*‐scores [20] and created a population set of 19,383 PubMed records on April 1, 2022 using the rentrez package [21] (see code availability statement for more details). This random sample is updated regularly.

### 2.3 Software development

We adopted a flexible and incremental approach to software development, with the first goal being to develop a MVP as quickly as possible for the defined use cases (Section 2.1 Use Cases). The MVP should be able to replace the existing workflow in Wordstat. To avoid duplication of effort and to build on existing solutions for text analysis and bibliographic formats in R, we searched the CRAN Task View “Natural Language Processing” [22, 23], the Systematic Review Toolbox [24, 25], the ESMAR Conference website [16], and the ESHackathon website [26]. We also checked the lists of R packages presented in the Utrecht Summer School course “Introduction to Text Mining with R” as well as the preliminary results of the EAHIL Evidence‐based Information Special Interest Group project “R for health libraries.” Finally, we conducted an exploratory Google search. The last search was conducted in May 2023. Please refer to the supplementary material for more details. The R package searchbuildR is currently released under a GPL 3.0 licence. To make the shiny app internally available to non‐coding information specialists, we set up a non‐public Linux‐based shiny server (version v1.5.20.1012) to allow direct access in a web browser, without the need to download or run the R code locally.

## 3 SOFTWARE FUNCTIONALITY

SearchbuildR works as a shiny app, which is called up with the function searchbuildR::run_app(). The text analysis process is implemented in 7 tabs: (1) “data import,” (2) “freetext,” (3) “MeSH,” (4) “qualifier,” (5) “all keywords,” (6) “free‐text terms in context,” and (7) “phrases.” Each tab and its underlying analysis is described in the following sections. All displayed tables can be filtered with a basic search. Table 1 gives an overview of all core features.

**Table 1:** Summary of all core features of searchbuildR.

Feature	Description
Data import	A test set is uploaded as a RIS format file (PubMed or Endnote).
Development and validation set generation	The test set is randomly split into a development set and a validation set in the ratio 2:1. PubMed identifiers (PMIDs) are provided as output in OVID search syntax.
Text analysis of free text terms, MeSH terms and MeSH qualifiers	Text analysis tables are provided and can be downloaded in the Western European CSV file format. They contain the statistics for all free text terms in the development set, which support the user to curate a Boolean search.
Free‐text terms in context	The original context of user‐specified terms is provided in a table and the full title and abstract are displayed for a user‐selected record.
Display of phrases	A table of all 2‐word combinations in the testset and their frequency is provided.

### 3.1 Data import

To start the analysis, a record set in RIS format must be uploaded in the “Upload a test set” section. The app currently accepts the RIS‐like PubMed format and the standard Endnote RIS export. The RIS tags in Table 2 are processed and displayed in the app. The uploaded records are displayed in a table with the columns “PMID,” “publication year,” “author,” and “title.”

**Table 2:** List of all RIS tags processed by searchbuildR.

RIS tags	Content
TY	Reference type
AN	Accession number (PMID)
AU or A1	Author
TI or T1	Title
AB or N1	Abstract
PY or Y1	Publication year
KW or DE	Keywords incl. MeSH terms
ER	End of reference

**Figure 1:**
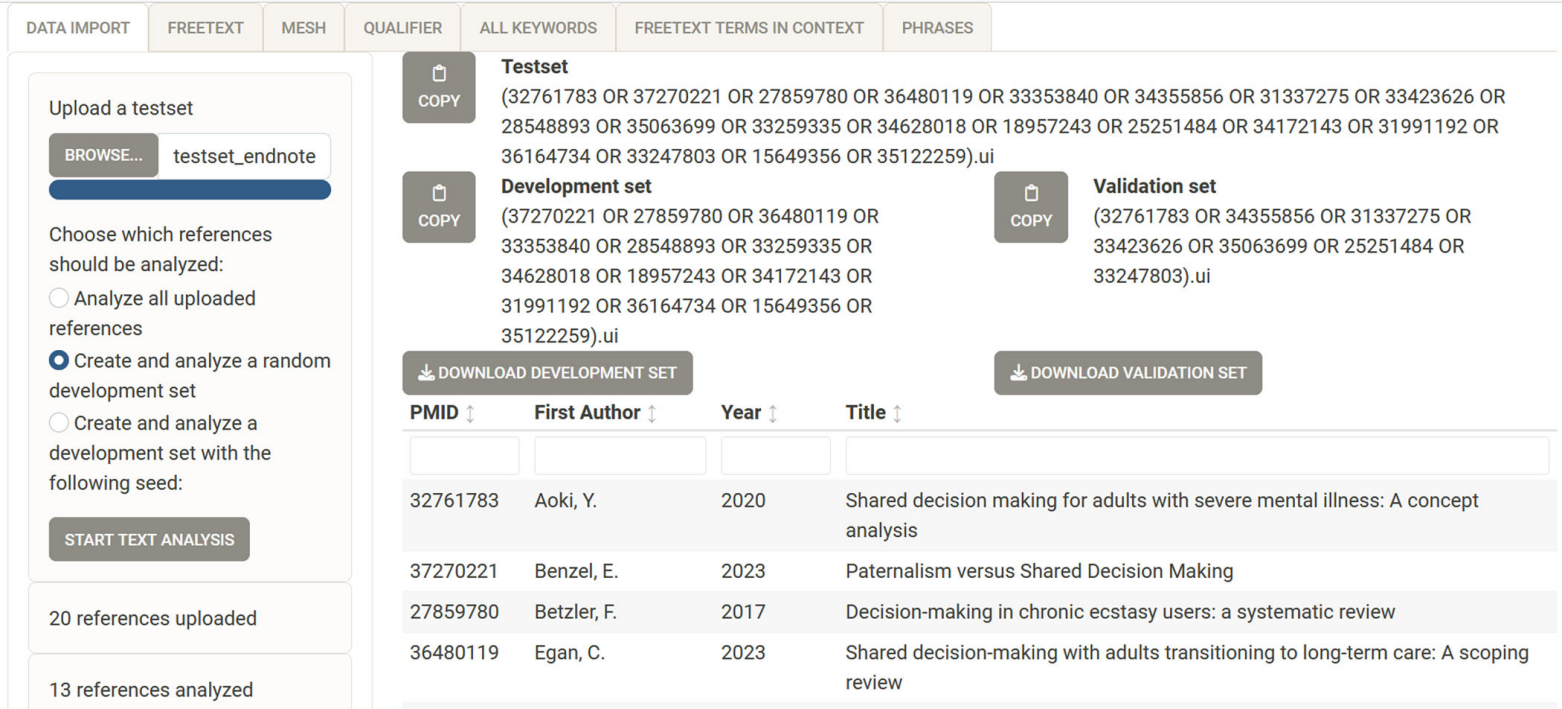
Screenshot of the start page (1) “data import” of searchbuildR after creation of a random development set from an uploaded test set of PubMed records.

In the “Choose which references should be analysed:” section, the user is offered three options for analysing the uploaded records: (1) All uploaded records. (2) The test set should be split into a development set and a validation set in a 2:1 ratio. Only the development set is analysed in the following tabs “free‐text,” “MeSH,” “qualifier,” and “all keywords.” The user can use the validation set to confirm the robustness of the results. After identifying potentially relevant search terms through the text analysis, the user should verify whether the validation set is also found with these terms. The app displays a random number (“applied random seed”), generated by R in the randomization process. At a later stage, this allows the user to reproduce the pseudo‐random process of creating the development and validation set. Option (3) is similar to (2), but provides more control over the creation of the development set by allowing the user to define the random number (“random seed”). This is a useful option for reproducing the same development and validation set from a given test set, for example in a peer review process. The selected option is confirmed with “start text analysis.” The PMIDs in OVID syntax for the development set, validation set, and all records are displayed separately and can be copied to the clipboard. There is also a download option for the full records in the development and validation set. The format is identical to the original upload format. They can also be imported into a reference management software for documentation purposes. Figure 1 shows an example output.

### 3.2 Frequency analysis

All frequency analysis tables in tabs (2), (3), and (4) have the following columns:

- “Candidate terms,” which can be searched for and sorted alphabetically.
- “Documents,” which shows the absolute number of records containing the candidate term/MeSH terms.
- “Documents in %,” which shows the relative number of records containing the candidate term/MeSH concepts.
- “*Z*‐Score,” which shows the result of the binomial test.
- “Term frequency,” which shows the absolute number of occurrences of the candidate term/qualifier. For MeSH terms, this column is skipped because the document frequency is equivalent to the term frequency, as they only occur once per record.

The results can currently be exported as a Western European CSV file (the value separator is a semicolon and the decimal separator is a comma).

#### 3.2.1 Free‐text

**Figure 2:**
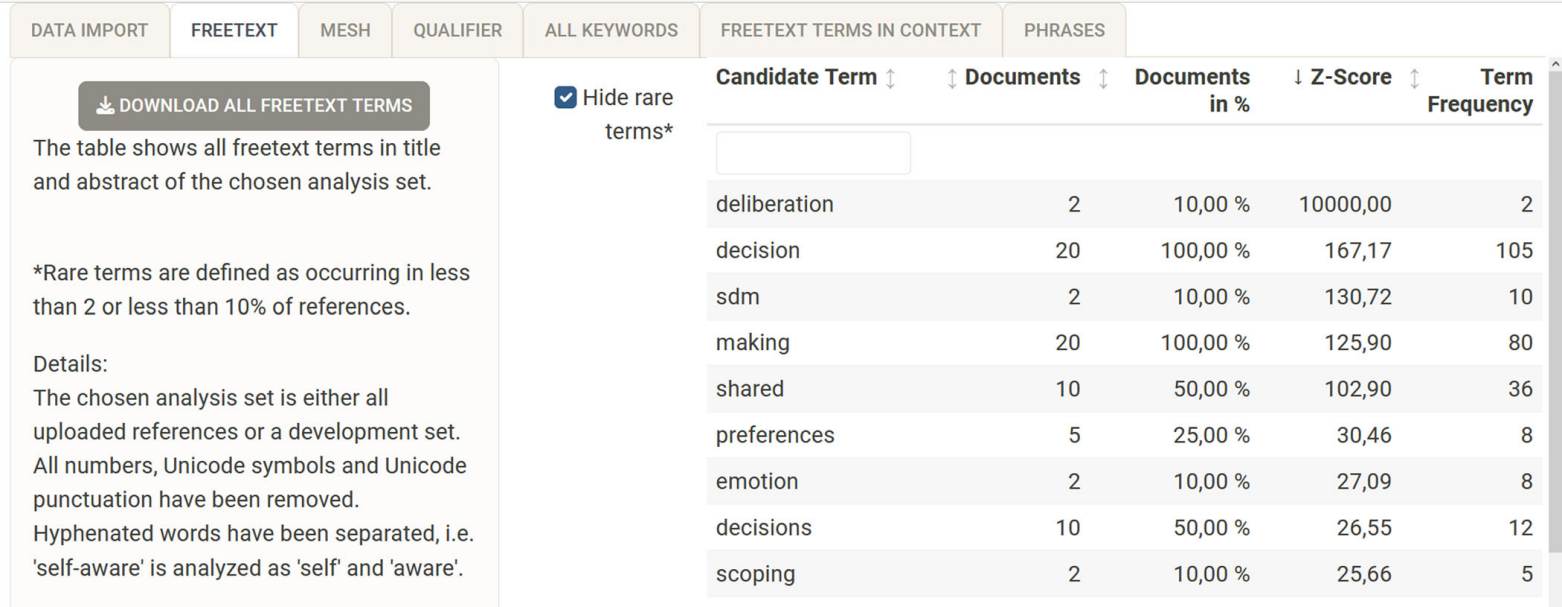
Screenshot of the searchbuildR tab (2) “freetext,” displaying the analysis results of each unique word of the titles and abstracts in the development set sorted by *z*‐scores in descending order.

Tab (2) “free‐text” displays the results of the frequency analysis and the *z*‐scores of the binomial test, as shown in Figure 2. All identified terms are displayed. A *z*‐score of 10,000 is assigned to all terms that do not occur in the population set. A term is defined as a word without hyphens, without Unicode symbols or Unicode punctuation, and is not a number. For example the hyphenated term, “self‐aware” would be analysed as the two terms “self” and “aware.” This decision was made in line with how most major search platforms (PubMed, Ovid, Wiley) and other textmining tools (e.g., PubReMiner) handle hyphenated terms, that is, hyphenated terms are treated as 2‐word combinations. A checkbox offers the possibility to hide rare terms, that is, terms appearing in only 1 record or in less than 10% of the total records.

#### 3.2.2 Controlled vocabulary: MeSH terms and qualifiers

**Figure 3:**
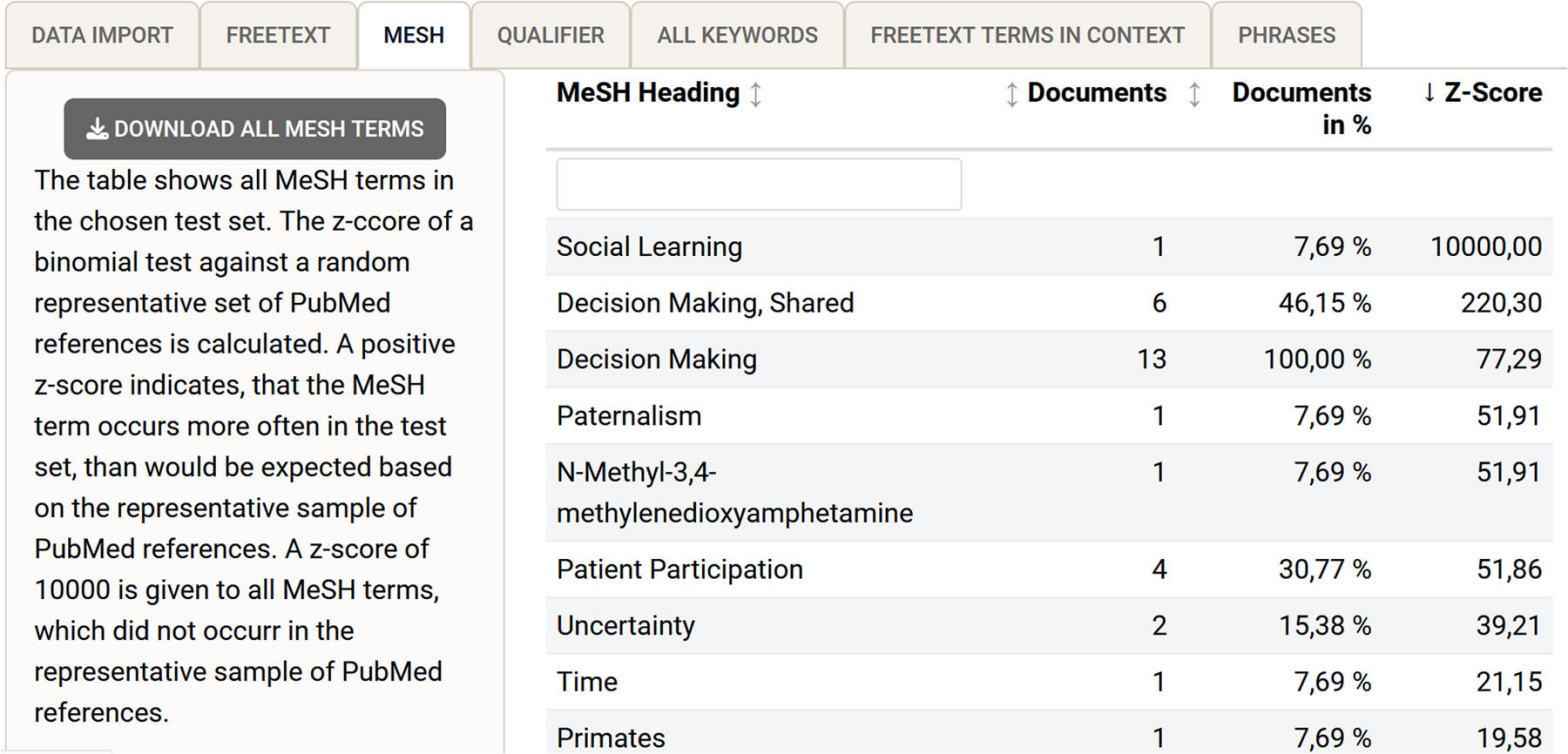
Screenshot of the searchbuildR tab (3) “MeSH,” displaying the analysis results of each unique MeSH heading in the development set sorted by z‐scores in descending order.

Tab (3) “MeSH” and tab (4) “qualifier” show the results of the frequency analysis of the MeSH vocabulary separately for MeSH terms and subheadings (=qualifiers); see Figure 3 for an example. MeSH subheadings occur only as a qualifying addition to a MeSH term, are less specific (e.g., “therapy”), and may occur more than once in a single record. Only terms included in the MeSH dictionary version of searchbuildR (currently 2022 MeSH XML) are displayed in these tabs, which may differ slightly from the latest available NLM online version.

### 3.3 All keywords

**Figure 4:**
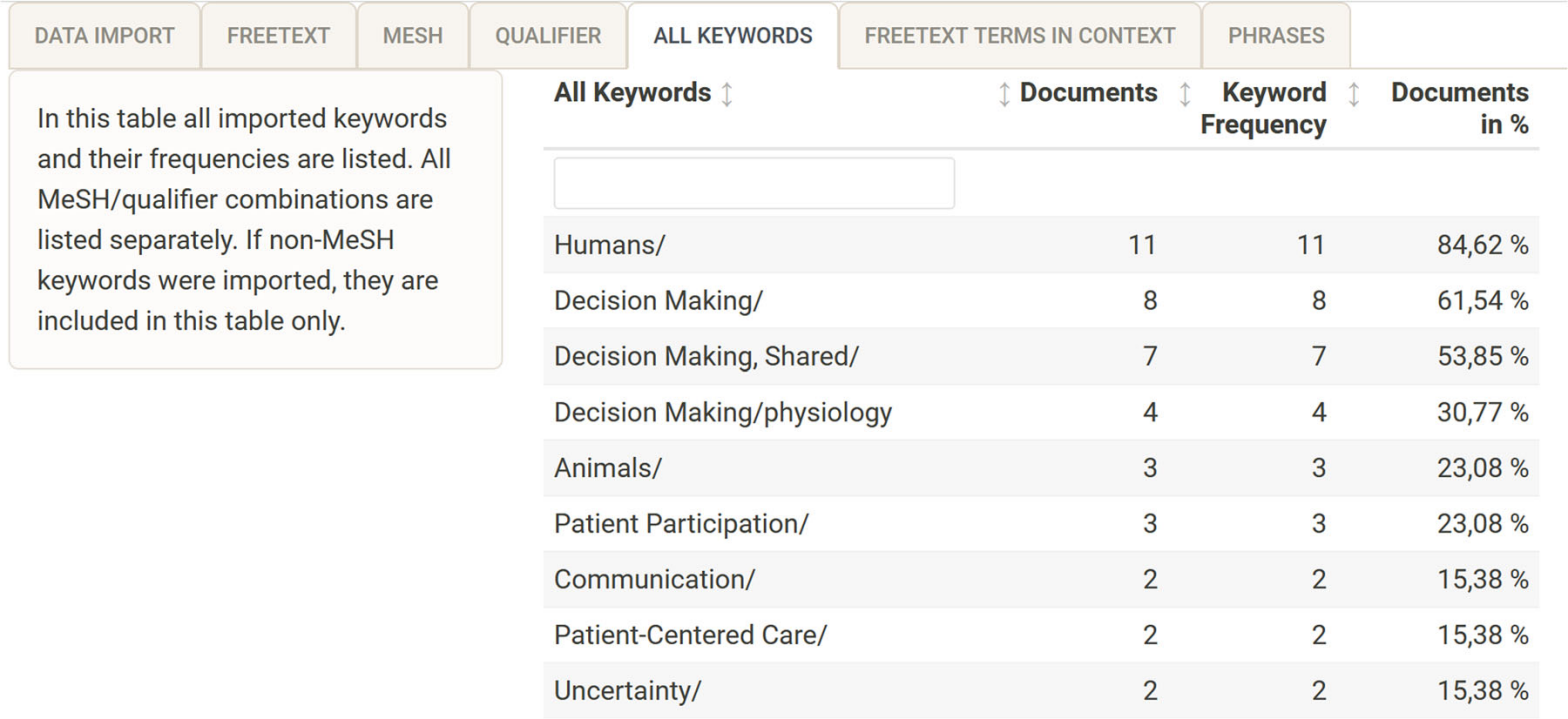
Screenshot of the searchbuildR tab (5) “all keywords,” displaying the analysis results of each unique imported keyword sorted by frequency in descending order.

Tab (5) “all keywords” shows everything that has been imported from the keyword tags. This includes MeSH/qualifier combinations, for example, “Neoplasms/diagnosis” and “Neoplasms/therapy” would be displayed separately in this table. Non‐MeSH terms from the keyword RIS‐tags may also be shown here. Absolute frequencies of unique keywords are shown, but no *z*‐scores are calculated (see Figure 4).

### 3.4 Free‐text terms in context

**Figure 5:**
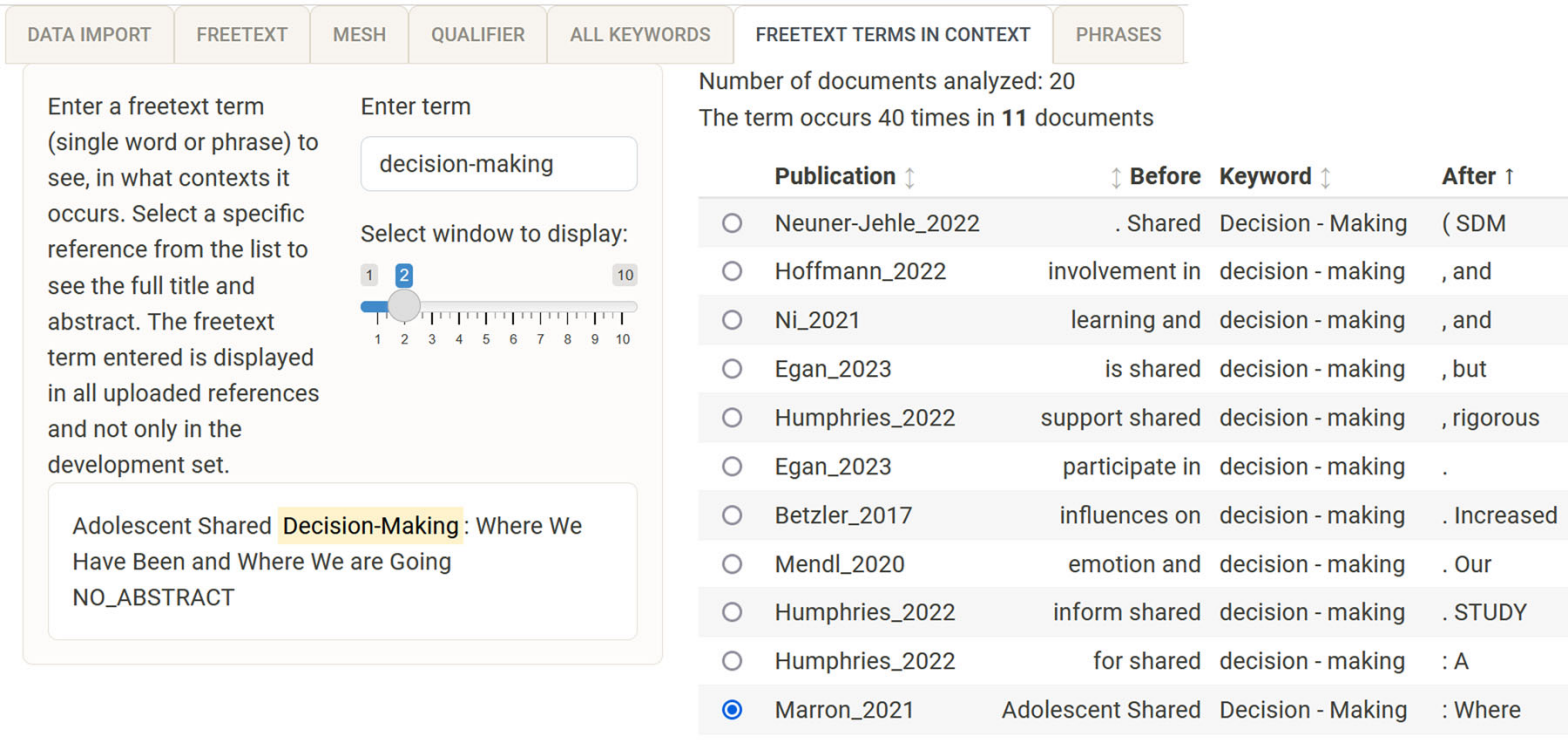
Screenshot of the searchbuildR tab (6) “freetext terms in context,” displaying the 2‐word window of each occurrence in the uploaded records for the term “decision‐making.” Further information is provided on the frequency of the term of interest in the test set. The complete text analysed for one record is shown for the selected record “Marron_2021.”

Tab (6) “free‐text terms in context” is an interactive tab that helps to understand the identified candidate terms in their original context. The user enters a search term of interest. Multiple words or hyphenated words are allowed (e.g., “breast cancer” or “decision‐making”) as shown in Figure 5. The title and abstract of a selected record are shown on the left of the panel. Blank titles or abstracts are replaced by “NO_TITLE” or “NO_ABSTRACT,” respectively. In the main panel on the right, all the occurrences of the entered term are displayed in their immediate context with the preceding and succeeding words. The word window can be customized by the user.

### 3.5 Experimental: Phrases

**Figure 6:**
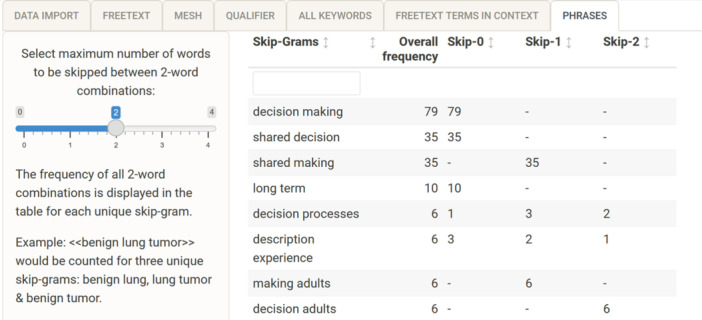
Screenshot of the searchbuildR tab (7) “phrases,” displaying a table of the frequency of 2‐word combinations separately for occurrences where 0, 1, or 2 words are skipped. The table is sorted by the overall frequency in descending order.

To develop a precise Boolean search strategy, it is often helpful to use proximity operators, that is, to search for terms that occur together in a defined word window. For example, to find the term “breast cancer” in “breast, ovarian or cervical cancer,” it makes sense to search for “breast” and “cancer” in proximity to each other rather than searching for a fixed quoted phrase. The “phrases” tab is a first approach to analysing two words and the distance between them in the test set. The tab shows all the 2‐word combinations identified, as well as the overall frequency and the specific frequency for each skip gram (i.e., a combination of words that skips a defined number of words) [27]. For instance, the term “shared making” would be a skip‐1 gram for the original term “shared decision making” of a given text; see Figure 6 for an example.

### 3.6 Software evaluation

**Figure 7:**
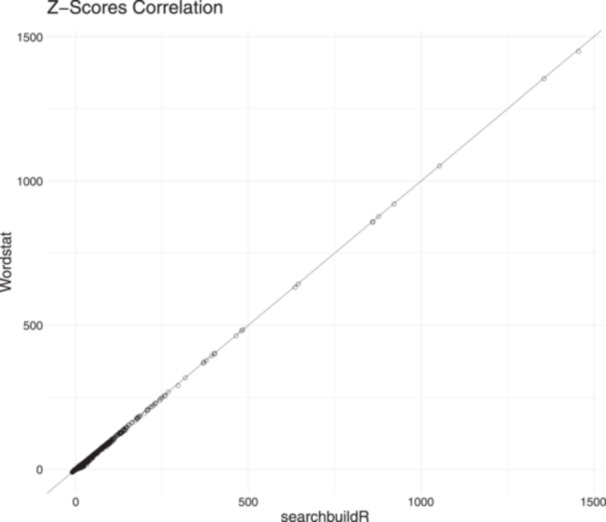
Scatterplot of the *z*‐scores calculated with searchbuildR and Wordstat.

We wanted to compare seachbuildR's *z*‐score ranking of candidate terms with that of Wordstat. We also evaluated user experience. We first developed a test suite for the package source code using the “testthat” package, as recommended by Wickham [28, 29]. Second, 3 test users tested searchbuildR (version 0.0.12) by checking the tool's usability and one test user reviewed the workflow prospectively in a new IQWiG report. Finally, equivalence to the previous implementation with Wordstat was tested the Pearson correlation of the resulting *z*‐scores is *r* > 0.99 and is presented in a scatterplot in Figure 7. The 3 test users checked the usability of the text analysis tool as well as the plausibility of the text analysis. Their review was discussed with the package developer and resulted in minor interface updates. To test equivalence, we compared the results of 10 published IQWiG projects. The test sets from these projects were re‐analysed in Wordstat with the latest population set and then analysed with the same population set in searchbuildR. The results are not intended to reproduce the process of search strategy development in the IQWiG projects. Rather, they serve as a realistic data set against which searchbuildR and Wordstat calculations can be compared. Details about the evaluation from version 1.0.0 are provided in the supplementary material. If the following requirements were met, this was considered defined as sufficient evidence to confirm the equivalence of the previous and new implementations:

(1) All terms identified by Wordstat are also identified by searchbuildR.
(2) All terms with a *z*‐score above 20 in Wordstat also have a *z*‐score above 20 in searchbuildR.
(3) A plausible explanation can be found for all differences in analysed terms between the searchbuildR results and the Wordstat results.

All requirements were met before searchbuildR was implemented as a new text analysis tool at IQWiG. The investigation of the differences in the analyses of terms showed that special characters (e.g., Greek letters) or units of measurement (e.g., micrometre, µm) are ignored by Wordstat, but not by searchbuildR. As these terms are not usually used in a search strategy, we consider these differences to be negligible for the assumption of equivalence between the two implementations of the objective approach.

## 4 DISCUSSION

SearchbuildR is designed as a tool to support the transparent and reproducible development of Boolean search strategies for systematic reviews of biomedical studies. It can identify the most relevant free‐text and MeSH terms in a set of bibliographic records. The randomization of the user's test set into a development and validation set and the text analysis are fully automated. SearchbuildR uses an approach to text analysis that relates the frequency of potential search terms to the overall occurrence of these terms in PubMed. With the *z*‐score, the package provides a metric that helps to rank the relevance of a term empirically, beyond its semantic meaning. This allows for a more objective selection (or omission) of search terms. In addition to single‐word search terms, searchbuildR provides features to examine words both in their immediate context and in 2‐word combinations. For experienced searchers, who tend to use more sophisticated techniques for searching bibliographic databases, the proximity in which two or more terms occur is essential information for developing concise and precise search strategies. Reproducibility is one of the goals of systematic reviews. SearchbuildR supports the process and documentation of query formulation in a way that is easily verifiable and reproducible by providing an objectively derived ranking of candidate terms from a given test set. Choosing which terms to include and combining search terms to build a Boolean search strategy are still manual steps. In comparison to other tools, searchbuildR provides a more meaningful ranking of candidate terms than PubReMiner or litsearchr. It is the only approach, that takes term occurrence in a database (Pubmed) into account by using a population set (see Hausner [3] for more details), and not only the frequency in the user provided test set. Some potential future features to improve the performance of searchbuildR are outlined below.

### 4.1 Planned features for future versions

#### 4.1.1 Truncation

A key concept in Boolean searches is truncating a term to search for multiple variations of a word at the same time. The simplest example is to truncate the plural “s” at the end of a noun (e.g., “neoplasm*” finds “neoplasms”). This option is not currently included in searchbuildR, but can easily be implemented with the quanteda [30] package.

#### 4.1.2 Phrases and skip grams

Another goal is to improve the performance of the “phrases” feature. The next step would be to rank the displayed 2‐word combinations in a more meaningful order than simply by the frequency of occurrence. For example litsearchr is specifically designed to identify phrases in a given test set using a sophisticated algorithm. However, it does not include skip grams, which are important for choosing proximity operators. We also aim to increase the number of phrases considered to multiple word combinations.

#### 4.1.3 Combining search terms for boolean searches

Another planned feature is to display the relative recall for combinations of search terms and phrases (e.g., “breast neoplasm OR tumour”) [31]. In the presented version 1.0.0, only the relative recall of single candidate terms is displayed. We have not implemented an approach to account for nonrelevant records, so neither precision nor accuracy can currently be estimated.

#### 4.1.4 Further potential features

Another desirable feature is missing in searchbuildR: As MeSH terms are organized in a tree structure, it would be useful to analyse all subordinate MeSH terms together (mimicking the “explode” search function for controlled vocabulary).

### 4.2 Limitations

While searchbuildR accelerates and supports the text analysis for search strategy development, it does not fully automate the process. First, while searchbuildR ranks free‐text and MeSH terms according to their relevance to the development set, it does not select any of these terms to build a search strategy. In addition, a subsequent comparison with the validation set can reveal further terms that were not present or not overrepresented in the development set (compare also to guidance in Hausner et al. [4]). Neither does searchbuildR suggest how to combine terms to build a Boolean search. While searchbuildR provides helpful outputs for all these tasks their execution remains the responsibility of the user. We are working on a tool that can suggest the optimal search strategy based on a test set of relevant records. However, there is still a long way to go. While some steps are automated, the evaluation and selection of each candidate term for building a complete search strategy are still an iterative and manual process.

## 5 CONCLUSION

IQWiG has developed and successfully tested the shiny app searchbuildR (latest version 1.0.0) to support the development of search strategies in systematic reviews. Further functions are being developed. It is open source and can be used by researchers and other information specialists without extensive R or programming skills. The package code presented in this report is openly available on GitHub at www.github.com/IQWiG/searchbuildR.

## AUTHOR CONTRIBUTIONS

**Claudia Kapp**: Conceptualization; data curation; formal analysis; methodology; project administration; resources; software; writing—original draft. **Naomi Fujita‐Rohwerder**: Software; writing—review and editing. **Jona Lilienthal**: Methodology; software; writing—review and editing. **Wiebke Sieben**: Methodology; writing—review and editing. **Siw Waffenschmidt**: Conceptualization; validation; writing—review and editing. **Elke Hausner**: Conceptualization; validation; writing—review and editing.

## PEER REVIEW

The peer review history for this article is available at https://www.webofscience.com/api/gateway/wos/peer-review/10.1002/cesm.12078.

## Supporting information

Supporting information.

Supporting information.

Supporting information.

Supporting information.

Supporting information.

Supporting information.

Supporting information.

Supporting information.

Supporting information.

Supporting information.

Supporting information.

Supporting information.

Supporting information.

Supporting information.

Supporting information.

Supporting information.

Supporting information.

## Data Availability

The data that supports the findings of this study are available in the supplementary material of this article. The package code presented in this report is openly available at GitHub at www.github.com/IQWiG/searchbuildR. The package version at the time of publication is 1.0.0.
