# Supplementary material for: The searchbuildR shiny app: A new implementation of the objective approach for search strategy development in systematic reviews
Source: Cochrane Evid Synth Methods. 2024 Jun 11;2(6):e12078. doi: 10.1002/cesm.12078 (PMC11795901; doi:10.1002/cesm.12078)
Supplement: Supplementary file 1 — Supporting information. [file CESM-2-e12078-s011.docx]

This README contains details on the provided supplementary material to the article „The searchbuildR shiny app: a new implementation of the objective approach for search strategy development in systematic reviews“.

**2-3_Software-Development.docx**

Supplementary information on the software development process complementing section 2.3 in the article.

Evaluation.txt

This document contains the R Code that was used for the evaluation. It is a quarto markdown document (.qmd)

**all_terms.csv, all_terms.txt**

The files contain data of all candidate terms included in the evaluation and the corresponding z-scores from searchbuildR and Wordstat.

A table with 4 columns ("Candidate term", "SearchbuildR Z-Score", "Wordstat Z-score", "Project"). Two formats are provided: comma separated values (csv-file) and tab separated values (txt-file). They are part of the original test results, which are referenced in the publication.

**terms_above_z_20.csv, terms_above_z_20.txt**

The files contain the data of candidate terms with a z-score >= 20 included in the evaluation and the corresponding z-scores from searchbuildR and Wordstat.

A table with 4 columns ("Candidate term", "SearchbuildR Z-Score", "Wordstat Z-score", "Project"). They are part of the original test results, which are referenced in the publication. Two formats are provided: comma separated values (csv-file) and tab separated values (txt-file). They are part of the original test results, which are referenced in the publication.

**Project1.txt - Project10.txt**

The raw RIS-format files, which were used for the text analysis.

***wordstat_list.R, wordstat_list.Rds***

Due to data format restrictions of the journal, the files containing data on the raw wordstat results are only available upon author request.
