# Supplementary material for: The searchbuildR shiny app: A new implementation of the objective approach for search strategy development in systematic reviews
Source: Cochrane Evid Synth Methods. 2024 Jun 11;2(6):e12078. doi: 10.1002/cesm.12078 (PMC11795901; doi:10.1002/cesm.12078)
Supplement: Supplementary file 2 — Supporting information. [file CESM-2-e12078-s013.docx]

Software Development

For basic R functions, we used the tidyverse framework [^1^](#_ENREF_1), a well-maintained and well-documented data science framework in R. For text mining and quantitative text analysis, we used the quanteda packages [^2^](#_ENREF_2). In addition, we used revtools [^3^](#_ENREF_3)^,^[^4^](#_ENREF_4) for handling bibliographic data and interactive data tables for R (“reactable” [^5^](#_ENREF_5) ) for displaying user-friendly tables in the shiny app. The full list of R packages that need to be installed to run searchbuildR is included in the metadata of the package (see the code availability statement for more details).

For the development process of the package, we followed the standards of Hadley Wickham (chief scientist at Posit PBC) [^6^](#_ENREF_6)^,^[^7^](#_ENREF_7), using the golem framework for production-grade shiny apps [^8^](#_ENREF_8). We used git for version control, as suggested by Jennifer Bryan [^9^](#_ENREF_9).

1. Wickham H, RStudio. tidyverse: Easily Install and Load the 'Tidyverse'. Accessed 30.11.2022, <https://CRAN.R-project.org/package=tidyverse>

2. Benoit K, Watanabe K, Wang H, et al. quanteda: An R package for the quantitative analysis of textual data. *Journal of Open Source Software*. 2018;3(30):774. doi:<https://doi.org/10.21105/joss.00774>

3. Westgate MJ. revtools: Tools to Support Evidence Synthesis. Accessed 08.01.2024, <https://cran.r-project.org/package=revtools>

4. Westgate MJ. revtools: An R package to support article screening for evidence synthesis. *Res Synth Methods*. 2019;10(4):606-614. doi:<https://doi.org/10.1002/jrsm.1374>

5. Lin G. reactable: Interactive Data Tables for R. Accessed 19.10.2023, <https://CRAN.R-project.org/package=reactable>

6. Wickham H, Bryan J. *R Packages*. 2023. <https://r-pkgs.org/>

7. Wickham H. *Mastering Shiny : build interactive apps, reports, and dashboards powered by R*. 2021. <https://mastering-shiny.org/>

8. Fay C, Rochette S, Guyader V, Girard C. *Engineering production-grade shiny apps*. 2022. <https://engineering-shiny.org/index.html>

9. Bryan J, Stat 545 TA, Hester J. *Happy Git and GitHub for the useR*. 2023. <https://happygitwithr.com/index.html>
